# Complete mitochondrial genome of the water vole, *Microtus richardsoni* (Cricetidae, Rodentia)

**DOI:** 10.1080/23802359.2020.1780640

**Published:** 2020-06-17

**Authors:** Fahad Alqahtani, Drew Duckett, Stacy Pirro, Ion I. Mandoiu

**Affiliations:** a Computer Science and Engineering Department, University of Connecticut, Storrs, CT, USA; b National Center for Artificial Intelligence and Big Data Technology, King Abdulaziz City for Science and Technology, Riyadh, Saudi Arabia; c Department of Evolution, Ecology and Organismal Biology, The Ohio State University, Columbus, OH, USA; d Iridian Genomes INC, Bethesda, MD, USA

**Keywords:** Water vole, complete mitochondrial genome, *Microtus richardsoni*, Cricetidae, Rodentia

## Abstract

Water voles (*Microtus richardsoni*) are sensitive species distributed in the mountains of Canada (Alberta, British Columbia), and the United States of America (Idaho, Montana, Oregon, Utah, Washington, and Wyoming). We assembled the complete circular *M. richardsoni* mitogenome, which is 16,285 bp in length and encodes 13 protein-coding genes, 22 tRNA genes, and two rRNA genes. We estimated the phylogenetic tree of *M. richardsoni* and 24 related arvicoline species with two outgroup species: *Phodopus roborovskii and Cricetus cricetus*.

Water voles (*Microtus richardsoni*) are considered sensitive species by the USDA Forest Service because of their small populations and specific habitat requirements. They are highly dependent on freshwater streams in the mountains of Canada (Alberta, British Columbia), and United States of America (Idaho, Montana, Oregon, Utah, Washington, and Wyoming) (Klaus et al. 2001).

We assembled the complete mitochondrial genome of a single *M. richardsoni* individual sampled from Hoodoo Lake, ID (46.333333 N, 114.633333 W) sequenced by Iridian Genomes, Inc. (Bethesda, MD; NCBI-SRA: accession number SRR8451905 and SRR8293759). Paired-end (2 × 150 bp) whole-genome sequencing (WGS) on an Illumina HiSeq X-Ten produced 186,970,325 and 117,371,326 read pairs, respectively. The percentage of mitochondrial read pairs in these libraries was estimated to be 0.91 and 1.63%, respectively. Moreover, the original specimen is in the Conner Museum at Washington State University, catalog number 04-1452.

We generated the mitogenome of M. richardsoni by using the SMART2 pipeline (Alqahtani and Mandoiu 2020). SMART2 was used to jointly assemble the two WGS libraries, additionally using a M. richardsoni cytochrome b gene sequence (GenBank ID AY973809) as seed sequence. The first step in SMART2 is automatic adapter detection and trimming. Second, SMART2 uses a doubling algorithm to automatically select the number of read pairs that are used to assemble the mitogenome. For *M. richardsoni*, the doubling algorithm selected 100,000 read pairs from each of the two WGS libraries. Next, SMART2 randomly resamples the selected number of trimmed read pairs from each library separately and filters the reads using a coverage-based filter based on the count distribution of the k-mers in the seed sequence. The fourth step is the preliminary assembly of reads passing the coverage-based filter using SPAdes (Bankevich et al. 2012). Fifth, preliminary contigs are filtered using BLAST (BLAST: Basic Local Alignment Search Tool 2019) searches against a local mitochondrial database. The sixth step is secondary filtering of the reads in the two libraries by alignment to preliminary contigs that have significant BLAST matches. The seventh step is a secondary de novo assembly using SPAdes for reads passing the alignment filter. Next, iterative scaffolding and gap filling are performed based on maximum likelihood. Lastly, mitochondrial genes are predicted and annotated using the MITOS annotation pipeline (Bernt et al. 2013). Steps 2-8 were repeated five times on randomly resampled sets of reads, generating an identical mitogenome sequence in all five runs (5/5 bootstrap support). Across both WGS libraries, the average number of mitochondrial reads assembled in each bootstrap sample was 3,527.8 ± 84.5884, with an average coverage is of 65×.

The mitogenome sequence (GenBank ID MT225016) generated by SMART2 has a length of 16,285bp. The mitogenome sequence annotation generated by MITOS has 13 protein-coding genes, 22 transfer RNA genes, and 2 ribosomal RNA genes. For our phylogenetic analysis, we downloaded 24 publicly available arvicoline mitogenomes and two outgroup sequences from GenBank and aligned them with our *M. richardsoni* mitogenome using the local pair algorithm in MAFFT (Katoh et al. 2002). The phylogeny was estimated using BEAST 2.5.0 (Bouckaert et al. 2019) with 10,000,000 Markov steps, 100,000 burn-in steps, and a GTR + I+G substitution model selected using jmodeltest (Posada 2008). The tree was visualized with FigTree (Rambaut and Drummond 2012). The phylogeny (Figure 1) shows *M. richardsoni* as sister to *M. ochrogaster* and paraphyly within the genus *Microtus*.

## Data Availability

Raw data is available in NCBI at https://www.ncbi.nlm.nih.gov/sra/ with accession numbers are SRR8451905 and SRR8293759. Also, the mitogenome sequence of *Microtus Richardsoni* is available in NCBI at https://www.ncbi.nlm.nih.gov/nuccore with accession number MT225016. Phylogenetic tree based on the complete mitochondrial genomes of 25 arvicoline species and two outgroups (*Cricetus cricetus* and *Phodopus roborovski*). The scale bar represents the number of substitutions per site and node labels represent posterior probabilities.
